# Psychotropic medication and in-hospital falls in older adults: a cohort-based secondary analysis with exploratory stratification among users

**DOI:** 10.1038/s41598-025-31320-7

**Published:** 2025-12-06

**Authors:** Maosong Wang, Jifeng Peng, Jianxin Hu

**Affiliations:** 1 School of Health and Elderly Care, Shandong Women’s University, Jinan, 250300 Shandong People’s Republic of China; 2 School of Nursing and Health, Xi’an Innovation College of Yan’an University, Xi’an, 710100 Shaanxi People’s Republic of China

**Keywords:** Psychotropic medication, Falls, Risk factors, Inpatients, Second-hand analysis, Diseases, Health care, Medical research, Risk factors

## Abstract

People using psychotropic medication have a higher risk of falls in the hospital environment. This study aims to investigate and assess the risk of falls in the people using psychotropic medication in hospitals. A secondary analysis was conducted to study and analyze the factors related to the risk of falls in the people using psychotropic medication collected from the Department of Health and Preventive Medicine in Fukushima City, Japan, between August 2008 and September 2009. Clinical records collected from structured questionnaires administered through face-to-face interviews conducted by nurses and doctors were used, and fall events were collected from the clinical records. Univariate and multivariate regression analyses were used to evaluate fall risk factors. ROC curves were used for predictive analysis of factors influencing falls. A total of 606 patients using psychotropic medications, of whom 43 (7.1%) experienced falls. Among the remaining 8863 patients, 187 (2.1%) experienced falls, and there was a significant difference between the two groups (*P* < 0.001). Multivariate regression analysis showed that age, eyesight, and admission days were independent risk factors for falls (all *P* < 0.05). The Model constructed with 11 indicators showed a good ROC curve (AUC = 0.811), with a sensitivity of 90.48% and specificity of 64.86%. Patients receiving psychotropic medications had a higher risk of in-hospital falls than those not receiving such medications. Within this subgroup, a multivariable model incorporating age, planned surgery, ADL (evacuation), and length of stay was used to quantify the association and showed acceptable internal performance; we suggest interpreting the model as an analytical aid.

**Supplementary Information:**

The online version contains supplementary material available at 10.1038/s41598-025-31320-7.

## Introduction

With the continuous improvement of human medicine and health levels, many countries are experiencing population aging, particularly in East Asian countries such as Japan, South Korea, and China^1–3^. As a result of population aging, the health issues of the older inpatients are receiving increasing attention from society and the medical community^4^. Falls are frequent in older adults and constitute a major risk factor in this population^5^. Falls not only cause injuries and illnesses among older inpatients but also increase the occurrence of complications and even lead to death^6^. Data shows that over 28% of older adults worldwide experience falls each year, with over 300 million seeking medical treatment due to falls and even over 680,000 older people dying due to falls^7^. Research has shown that in China alone, at least 20 million older people experience adverse fall events during hospitalization, resulting in direct medical costs of over 5 billion RMB^8,9^. Falls among older adults lead to both individual suffering and societal burden.

A survey conducted in Japan involving approximately 9957 older inpatients individuals over about one year investigated the risk factors for hospital falls^10^. The results showed that the proportion of falls among the older inpatients reached 2.5%, and the incidence was 3.28 per 100 person-days. Their multivariate regression analysis on the risk of falls revealed that age, history of falls, and the need for assistance in activities of daily living (ADL) were common risk factors for falls in the study population. The study suggested that age, history of falls, and ADL assistance needs should be the most crucial information obtained when patients are admitted. Hospitals should consider incorporating corresponding fall prevention care plans based on the research findings into their daily management. In the study by Takehito Hayakawa et al., the odds ratio (OR) for falls associated with the use of Psychotropic medication in the male population was as high as 3.59 (2.00, 6.47), with *P* < 0.05. When the study’s male-to-female ratio was close to 1:1, there might be a higher probability of falls in the overall population using Psychotropic medication.

Several studies have shown that falls are a common accidental injury among psychiatric inpatients^11–13^. Research has indicated that the fall incidence rate among older inpatients psychiatric inpatients can reach 25% within three years, and these falls can result in head injuries or fractures, negatively impacting the patients’ recovery process^14^. Long-term medication of psychiatric patients can lead to various symptoms of central nervous system impairment^15^. As they age, their organ functions deteriorate more rapidly than the general population. Additionally, due to the prolonged hospitalization and limited physical activity of psychiatric patients, their balance abilities are compromised, increasing the risk of falls^16^. Fabian Wedmann et al. conducted a study involving 481 older individuals who experienced falls in the hospital. The research revealed a significant correlation between medications, including serotonin-noradrenaline reuptake inhibitors and psychotropic drugs, and falls among hospitalized patients^17^. In a retrospective case–control study, Lindgren de Groot et al. found that inpatients who fell were more likely than non-fallers to be receiving several classes of psychotropic and neurologic medications—such as antidepressants, antipsychotics, anxiolytics/hypnotics, and antiepileptics—suggesting an association between these medications and increased fall risk^18^. However, a Literature Review by Sarah Laberge and colleagues, encompassing 25 studies, indicated an association between benzodiazepines and antidepressant medications and the risk of hospital patient falls. Conversely, antipsychotic drugs, antihypertensive medications, antiepileptic drugs, and alcohol showed no correlation with the risk of hospital falls^19^.

During a systematic search of studies on inpatient falls, we noted that fall incidence and risk differed among inpatients receiving psychotropic medications. Although the study by Takehito Hayakawa et al. reported various circumstances leading to in-hospital falls, it did not provide a dedicated, in-depth description and analysis of psychotropic medication use. Given the sufficient sample size and well-documented variables in the present dataset, we therefore conducted a secondary analysis focusing on psychotropic medication use during hospitalization. We aimed to quantify its independent association with in-hospital falls and to explore potential effect modifiers and key risk factors, thereby providing more substantial evidence to inform clinical risk stratification and fall-prevention strategies. To address the key uncertainty, our primary aim is to demonstrate with convincing data whether in-hospital exposure to antipsychotic medications independently increases the risk of inpatient falls among older adults. As a practical implication of this analysis, the findings aim to inform staff awareness and guide hospital practices in preventing in-hospital falls.

## Materials and methods

### Data source

Dryad is an open-source, community-driven project that embraces a unique methodology for digital publication and data preservation. The original version of Dryad was launched in 2009 and built using the open-source DS pace repository software. In 2019, Dryad merged with Dash, a data publication service developed at the University of California Curation Center, a program operating within the California Digital Library. Dryad strongly emphasizes search, presentation, and discovery while delegating the responsibility of data preservation to the integrated underlying repository.

We conducted a retrospective cohort study using data from the Dryad database. According to the terms of service of Dryad, we cited the Dryad data package (“Data from Risk factors of falls in inpatients and their practical use in identifying high-risk persons at admission, Fukushima Medical University Hospital cohort study,” Dryad, Dataset, 10.1136/bmjopen-2014-005385). In this study, hospitalized patients obtained informed consent to participate in the initial research at admission. Therefore, this study did not require obtaining informed consent from the participants included in the study. This study did not involve approval or consent from an ethics committee.

### Study design and selection of study sample

A secondary analysis was conducted to analyze the risk factors for falls in the population using psychotropic medication collected by the Department of Public Health and Preventive Medicine at Fukushima Medical University, Japan, from August 2008 to September 2009. The source study from which the present dataset was obtained had been approved by Fukushima Medical University (approval registration No. 726), with informed consent obtained as applicable. Clinical records for the study were obtained from structured questionnaires administered through face-to-face interviews with the participants by nurses and doctors. Fall events were collected from the clinical records. The inclusion criteria for the study were: (1) individuals with records of psychotropic medication use in the dataset and (2) availability of detailed clinical records and baseline data regarding falls. The exclusion criteria were: (1) individuals with unknown psychotropic medication use, (2) individuals without detailed records of falls and clinical data, and (3) participants lost to follow-up.

### Variables

The basic information of the included population consisted of gender (Male vs. Female), age, wheelchair use (Yes vs. No), needs help to move (Yes vs. No), rehabilitation status (Yes vs. No), use of laxative medication (Yes vs. No), use of a remote carrying system (No vs. Crip censor vs. Other crip censors), presence of cognitive dysfunction (Yes vs. No), eyesight (no vs. use glasses vs. need help one vs. need help two vs. trance), use of sedative medication and hypnotic medication (Yes vs. No), use of a censor for the bed (Yes vs. No), planned surgery (Yes vs. No), history of falls (Yes vs. No), ADL-related indicators (normal vs. need help), lower extremity assessment using the Manual Muscle Test (MMT) (normal vs. abnormal), and length of hospital stay, among others. Participants were asked about their ability to independently perform seven ADL: standing, sitting, dressing, eating, toileting, evacuation, and washing their faces. The need for assistance in any of these activities indicated a low level of ADL. Subjects were evaluated using the MMT, with impairment defined as MMT < 4.

### Statistical analysis

This study’s data analysis was performed using SPSS 25.0 software. The count data between the two groups were compared using the chi-square test. For continuous data that followed a normal distribution, pairwise comparisons were performed using the t-test. Non-parametric tests (Kruskal–Wallis H test) were used for comparing continuous data that did not follow a normal distribution between the two groups. The Wilcoxon rank-sum test was employed for handling ordinal data. Risk factors for falls in the population were assessed using logistic regression analysis. The logistic regression analysis was performed in two steps. In the first step, univariate regression analysis was conducted to examine the risk indicators. We assessed multicollinearity using variance inflation factors (VIF), adopting VIF < 10 as the acceptable threshold. For multivariable modeling, we prespecified the covariate set based on prior evidence and clinical plausibility, rather than selecting variables by univariate *P*-values (*P* < 0.10). We applied LASSO regression to screen candidate variables further, thereby reducing potential bias and limitations arising from subjective variable selection or methodological constraints in logistic regression. The predictive ability of the risk indicators with statistical differences in the multivariate regression analysis was evaluated using ROC curves. In this study, a *P*-value less than 0.05 was considered statistically significant.

## Results

### Basic information about the included population

Takehito et al.‘s original data showed that detailed data from 9469 hospitalized individuals were included in this study from August 2008 to September 2009. Among them, 606 patients used psychotropic medication, and 43 cases of falls occurred during the study period (7.1%). Among the remaining 8,863 patients, there were 187 cases of falls (2.1%), and there was a significant difference between the two groups (χ^2 = 59.5, *P* < 0.001). There were statistical differences between the psychotropic medication user group and the non-user group in terms of gender, age, length of hospital stay, wheelchair use, needs help to move, cognitive function, sedative medication, hypnotic medication, planned surgery, ADL related items, and MMT of the lower limbs. For detailed results, please refer to Supplementary Table 1.

In the psychotropic medication group, the median age of the fallers was 70.0 (52.0, 77.0) years, while the median age of non-fallers was 57.0 (37.0, 72.0) years, and there was a significant difference between the two groups (*P* = 0.002). There were statistical differences between the two groups in terms of admission days, wheelchair use, needs to help move, planned surgery, history of falls, ADL-related items (clothes and wash face), and MMT of the lower limbs (all *P* < 0.05). However, the two groups had no statistical differences regarding gender, rehabilitation, use of remote carrying system, cognitive dysfunction, eyesight test, laxative medication, sedative medication, hypnotic medication, and other ADL assessments (all *P* > 0.05). For detailed results, please refer to Table 1.

**Table 1:** Baseline data analysis of the risk of falls in the psychotropic medication inpatient population.

Indicator	Groups	No (*n* = 53)	Yes (*n* = 43)	X^2^/z	*P*
Gender	Male	213	20	1.271	0.260
	Female	350	23		
Age		57.0 (37, 72.0)	70.0 (52.0, 77.0)	3.099	**0.002**
Wheelchair	No	430	23	11.089	**0.001**
	Yes	133	20		
Needs help to move	No	458	25	13.303	**< 0.001**
	Yes	105	18		
Rehabilitation	No	539	40	3.609	0.167
	Yes	6	2		
Laxative medication	No	348	22	1.905	0.195
	Yes	215	21		
Remote carring system	No	555	42	1.753	0.487
	Crip censor	5	1		
	Other crip censor	3	0		
Cognitive dysfunction	No	519	37	1.260	0.262
	Yes	44	6		
Eyesight	No	302	26	2.683	0.548
	Use glasses	220	16		
	Need help 1	26	0		
	Need help 2	13	0		
	Trance	2	0		
Sedative medication	No	541	40	0.951	0.329
	Yes	22	3		
Hypnotic medication	No	243	18	0.028	0.868
	Yes	320	25		
Censor of bed	No	555	42	0.223	0.636
	Yes	8	1		
Rihabiritation reviesed valuable	No	539	40	0.808	0.369
	Yes	23	3		
Inhibition	No	528	41	0.171	0.679
	Yes	35	2		
Planned surgery	No	393	36	4.478	**0.034**
	Yes	165	6		
History of falls	No	457	26	10.991	**0.001**
	Yes	104	17		
ADL clothes	Normal	435	26	6.768	**0.009**
	Need help	128	17		
ADL wash face	Normal	435	26	6.194	**0.013**
	Need help	128	17		
ADL evacuation	Normal	429	27	3.856	0.050
	Need help	134	16		
MMT Right foot	Normal	486	31	6.846	**0.009**
	Abnormal	75	12		
MMT left foot	Normal	500	31	11.226	**0.001**
	Abnormal	60	12		
Admission days		11.0 (5.0, 22.0)	26.0 (12.0, 42.0)	4.245	**< 0.001**

ADL, activity of daily living; MMT, manual muscle test.

Significant values are in [bold].

### Regression analysis of factors influencing falls

**Fig. 1:**
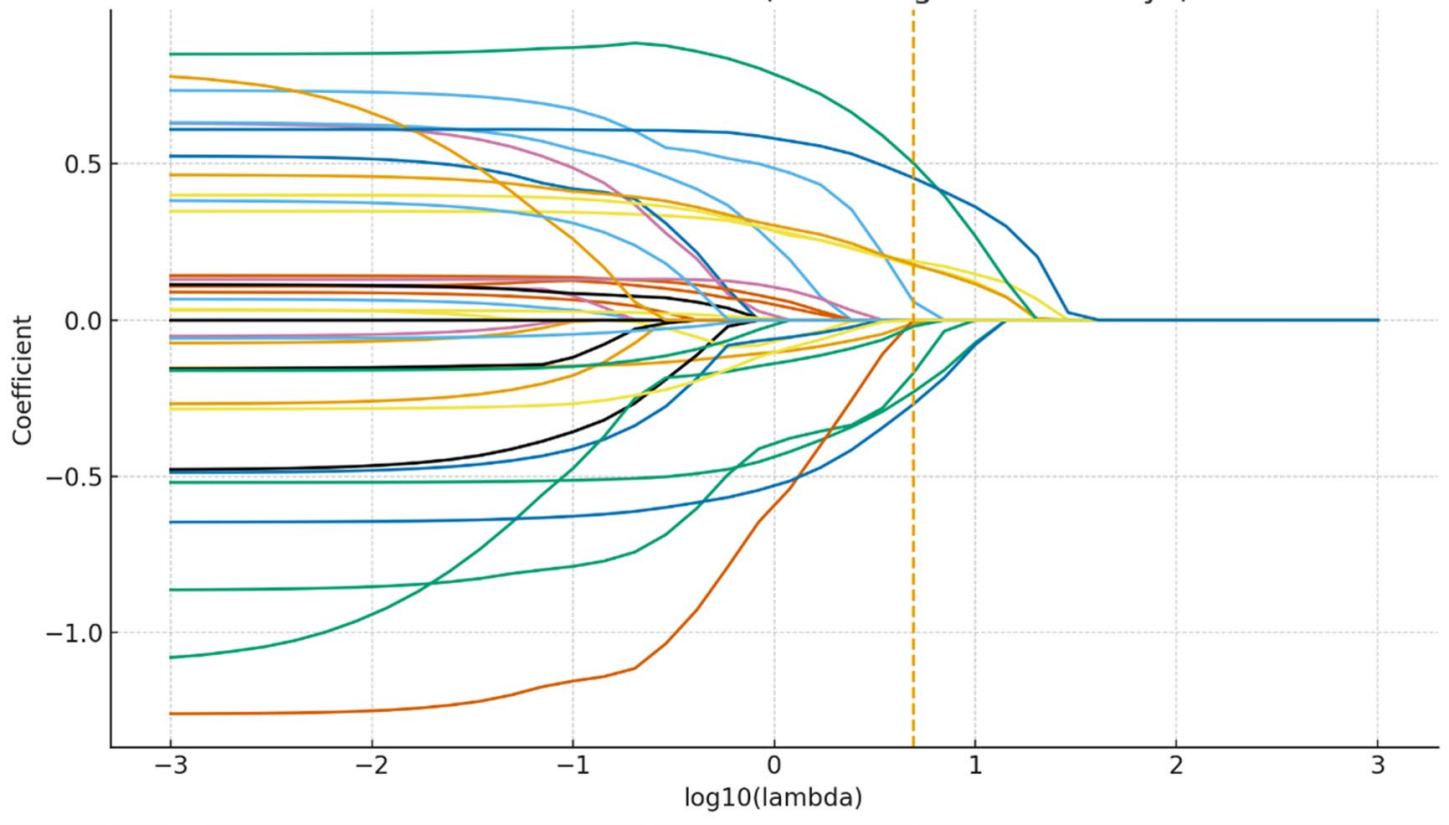
LASSO coefficient paths.

The results of the univariate regression analysis showed that among the 18 included indicators, 10 indicators had a result value < 0.1. Based on clinical experience, we ultimately included 15 variables in the logistic regression analysis. Subsequently, we conducted a multivariate regression analysis, and the results showed that age [OR, 1.042 (1.019, 1.066)]), admission days [OR, 1.031(1.019, 1.044)], and eyesight [OR, 454 (0.236, 0.873)] were independent risk factors for falls in the psychotropic medication group (Model 1, all *P* < 0.05). The LASSO analysis identified 10 predictors (Fig. 1; Table 2). Among them, age [OR, 1.056 (1.021, 1.093)], planned surgery [OR, 0.328 (0.130, 0.829)], admission days [OR, 1.030 (1.015, 1.045)], and ADL feeding [OR, 0.551 (0.349, 0.870)] were independent factors associated with in-hospital falls (Model 2, all *P* < 0.05). For detailed results, please refer to Table 3.

**Table 2:** Predictors with non-zero coefficients in the LASSO-penalized logistic regression model for in-hospital falls.

Predictor	Description	Standardized coefficient (β)*	Direction†
Age category	Age category	0.5025	Positive (↑ risk)
Admission days	Length of hospital stay (days)	0.4550	Positive (↑ risk)
Planned surgery	Planned surgery at admission	− 0.2303	Negative (↓ risk)
Eyesight	Visual function score	− 0.2693	Negative (↓ risk)
Needs help to move	Need help to move/transfer	0.1885	Positive (↑ risk)
History of falls	History of previous falls	0.1734	Positive (↑ risk)
MMT Left foot Group	Left foot muscle strength (grouped)	0.1779	Positive (↑ risk)
ADL Feeding	Feeding ability (ADL item)	− 0.1695	Negative (↓ risk)
ADA clothes Group	Dressing ability (ADL item, grouped)	0.0587	Positive (↑ risk)
Sex	Sex	− 0.0101	Slight negative

*All predictors were standardized (mean = 0, SD = 1) before LASSO estimation; coefficients are penalized logistic regression estimates at the optimal penalty selected by 5-fold cross-validation.

†Direction indicates the sign of the coefficient with respect to the log-odds of in-hospital falls.

**Table 3:** The analysis results of the univariate and multivariate regression model.

Indicator	Univariate analysis		Multivariate analysis (Model 1)		Multivariate analysis (Model 2)	
	OR	*P*	OR	*P*	OR	*P*
Age	1.028 (1.009, 1.047)	**0.004**	1.042 (1.019, 1.066)	**< 0.001**	1.056 (1.021, 1.093)	**0.002**
Gender	0.700 (0.375, 1.305)	0.262	0.688 (0.339, 1.395)	0.300	0.687 (0.347, 1.359)	0.246
Wheelchair	2.811 (1.497, 5.249)	**0.001**	1.077 (0.386, 3.002)	0.887		
Needs help to move	3.141 (1.653, 5.967)	**< 0.001**	2.374 (0.815, 6.917)	0.113	1.897 (0.784, 4.592)	0.156
Planned surgery	0.397 (0.164, 0.960)	**0.040**	0.392 (0.151, 1.016)	0.054	0.328 (0.130, 0.829)	**0.018**
History of falls	2.873 (1.504, 5.489)	**0.001**	2.045 (0.945, 4.425)	0.069	1.939 (0.921, 4.082)	0.081
ADL clothes group	2.283 (1.208, 4.312)	**0.011**	1.013 (0.599, 1.714)	0.962	0.971 (0.387, 2.437)	0.951
ADL wash face	2.222 (1.169, 4.224)	**0.015**	0.806 (0.457, 1.421)	0.456		
ADL evacuation	1.897 (0.992, 3.627)	0.053		–		
MMT right foot	2.508 (1.234, 5.009)	**0.011**	1.177 (0.624, 2.223)	0.615	1.348 (0.576, 3.158)	0.491
MMT left foot	3.226 (1.573, 6.615)	**0.001**	0.815 (0.432, 1.536)	0.526		
Admission days	1.031 (1.019, 1.044)	**< 0.001**	1.031 (1.016, 1.047)	**< 0.001**	1.030 (1.015, 1.045)	**< 0.001**
Laxative medication	1.545 (0.830, 2.877)	0.170				
Cognitive dysfunction	1.913 (0.765, 4.780)	0.165	0.875 (0.273, 2.811)	0.823	0.777 (0.254, 2.374)	0.658
Eyesight	0.638 (0.372, 1.095)	0.103	0.454 (0.236, 0.873)	**0.018**		
Sedative medication	1.844 (0.529, 6.428)	0.337	1.312 (0.259, 6.654)	0.743		
Hypnotic medication	1.055 (0.563, 1.977)	0.868	1.302 (0.630, 2.690)	0.475		
ADL Feeding	0.971 (0.747, 1.262)	0.826	–	–	0.551 (0.349, 0.870)	**0.011**

Model 1: presents the results of the multivariable regression analysis, which included variables with *P* < 0.1 from the univariable analysis, as well as clinically relevant variables with *P* > 0.1 retained based on clinical experience. Model 2: The model presents the results of a logistic regression analysis based on eight predictors selected by LASSO.

OR, odds ratio; ADL, activity of daily living; MMT, manual muscle test.

Significant values are in [bold].

### Predictive value of independent factors for falls

We conducted a predictive analysis using ROC curves for the four independent factors contributing to falls. The results showed that the four indicators had a low diagnostic value, with AUCs ranging from 0.567 to 0.694 (Table 4). Among the four indicators, Admission days had the most considerable AUC value (AUC = 0.694), with a sensitivity of 67.44% and specificity of 68.33%. The inflection point for falls in the population occurred on the 18th day. Although there were statistically significant differences between Admission days and ADL evacuation or planned surgery (*P* < 0.05), these differences provided limited diagnostic assistance for fall prediction. Age had a modest predictive value with an AUC of only 0.642, sensitivity of 62.79%, and specificity of 64.83%. The inflection point for falls in the population occurred at 65 years old. Planned surgery (AUC = 0.576) had a poor diagnostic value, while ADL evacuation had almost no diagnostic value (*P* = 0.081).

**Table 4:** The results of ROC curves for the independent influences of inpatient falls and the constructed models in multivariate regression analysis.

Indicator	AUC	SE	95% CI	Sensitivity (%)	Specificity (%)	AC	Youden	z-statistic	*P*
Age	0.642	0.045	0.602–0.680	62.79	64.83	> 65	0.276	3.139	**0.002**
ADL evacuation	0.567	0.038	0.527–0.607	37.21	76.20	> 0	0.134	1.748	0.081
Plan surgery	0.576	0.029	0.536–0.616	85.71	29.57	≤ 0	0.153	2.637	**0.008**
Admission days	0.694	0.047	0.655–0.730	67.44	68.33	> 18	0.358	4.091	**< 0.001**
Model 1	0.800	0.035	0.766–0.832	82.93	64.13	> 0.047	0.471	8.578	**< 0.001**
Model 2	0.796	0.039	0.761–0.828	73.81	76.85	> 0.074	0.507	7.621	**< 0.001**

ROC, receiver operating characteristic curves; AUC, area under curve; SE, standard error; 95% CI, 95% confidence interval; AC, Associated criterion; Youden, Youden index J.

Significant values are in [bold].

**Fig. 2:**
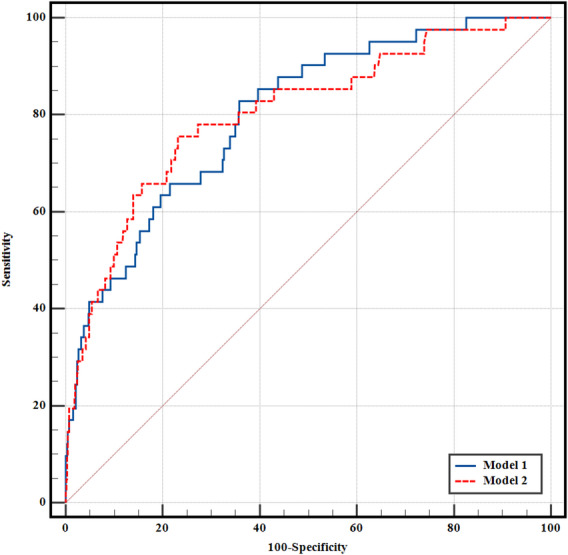
Diagnostic value of each indicator. Model 1: All 15 indicators included in the multivariate regression analysis were included; Model 2: LASSO logistic regression result. ROC: Receiver operating characteristic curves; AUC: Area under curve; ADL: Activities of daily living.

When we used the 15 indicators included in the multivariate regression to construct Model 1, the results showed that the Model achieved a moderate diagnostic level. The Model 1 had an AUC of 0.800, a sensitivity of 82.93%, and a specificity of 64.13%. The AUC of model 2 was 0.790, with a sensitivity of 73.81% and a specificity of 76.85% (Fig. 2). In terms of AUC, the diagnostic performance of the two models was very similar; Model 1 showed slightly higher sensitivity (82.93%), whereas Model 2 demonstrated a more balanced performance.

## Discussion

In our study, the fall rate among patients receiving psychotropic medications was 7.1% (43/606), compared with 2.1% (187/8863) in those not receiving such medications, with a statistically significant difference between the two groups (*P* < 0.05). Taken together with existing evidence, this pattern suggests that psychotropic medication use may be an important contributor to increased in-hospital fall risk in older patients. Within this psychotropic medication cohort, our findings further indicate that falls are not driven by a single dominant factor but by the combined influence of age, functional status, visual function, treatment plans, and hospitalization characteristics. In the conventional multivariable model (Model 1), older age and longer admission days consistently emerged as independent risk factors for hospital falls. At the same time, impaired eyesight was also significantly associated with falls, highlighting the joint impact of intrinsic vulnerability and prolonged exposure to the hospital environment. When LASSO-penalized regression was applied (Model 2), age and admission days remained robust predictors, and planned surgery and better ADL feeding function were associated with a lower risk of falls, suggesting that patients with preserved self-care ability and structured perioperative management may benefit from more effective monitoring and support. Notably, each predictor alone demonstrated only limited discriminative ability (AUC generally < 0.70), whereas combining multiple clinically plausible factors yielded moderate predictive performance (AUC ≈ 0.79–0.80), underscoring that fall risk assessment in older inpatients receiving psychotropic medications is best approached using an integrated, multidimensional model rather than reliance on any single variable.

Falls are influenced by individual health, disease, and environmental factors and can occur at any age, especially among older adults with higher incidence rates^20^. The rate of falls in older inpatients with psychiatric disorders is even higher, resulting in a more significant disease burden. According to a report from the Centers for Disease Control and Prevention (CDC) in the United States, one out of every four adults aged 65 or older experiences a fall yearly^21^.

Due to changes in mental state, unstable cognitive function, and side effects of psychotropic medication, falls among individuals with mental disorders have been increasing annually. Falls occur more frequently in older inpatients with mental disorders, compounded by age-related factors^22^, diseases, and hospitalization environments, resulting in higher fall rates. Research by Saulnier indicates that the risk of falls in older inpatients dementia patients is two to three times higher than in the general older inpatients^23^. In the study by Asada, older inpatients with depression, dementia, and long-term hospitalization, as well as those with schizophrenia, were particularly prone to falls^24^. A large-scale epidemiological study in Germany showed that the fall rate among older inpatients with cognitive impairments was 12.9%, compared to only 4.2% among older inpatients without cognitive impairments^25^.

Falls are a common health issue among older adults, with high incidence rates, severe consequences, and a significant disease burden, posing a serious global public health problem that threatens the physical and mental well-being of the older inpatients. With the aging population, the ability to regulate balance decreases, and various underlying diseases and decreased responsiveness are important risk factors for falls in older adults. Epidemiological studies have shown that one-third of individuals aged 65 or older worldwide experience falls yearly, increasing to 50% among those aged 80 or older. In this study, both models consistently demonstrated that increasing age is an independent risk factor for in-hospital falls among patients receiving psychotropic medications (both ORs >1, *P* < 0.05). Moreover, when the age was over 65, it became a risk inflection point for fall incidence in this population. Additionally, Model 2 showed that planned surgery was an independent influencing factor for fall risk in the population. This may be because individuals planning surgery have more serious physiological or pathological issues. In addition, admission days showed a similar trend to age in both models (both ORs >1, *P* < 0.05). Research by Dennis et al. showed that the fall incidence in geriatric psychiatric wards increased from 3.2 cases per 1000 hospital days to 17.1 cases per 1000 hospital days^26^, far exceeding the fall incidence reported in general psychiatric units by the International Quality Indicator Project (IQIP) (2.0 cases per 1000 hospital days). The instability of the physical condition of the population dependent on psychotropic medications and the side effects of these medications significantly contribute to the increased risk of falls in older adults.

Most factors influencing falls among older adults can be modified, and early identification and intervention in these risk factors can effectively reduce the incidence of falls^27^. Additionally, using scientific and accurate fall assessment tools is crucial for identifying fall risk factors^28^. However, further research is needed to confirm this conclusion through more related studies. The study on the risk of falls associated with psychotropic medication use aligns with the initial study by Takehito Hayakawa et al.^10^ indicating that age and fall history are risk factors for recurrent falls in the population. However, our study further demonstrated no gender difference among the factors that increase the risk of falls due to psychotropic medication use, and ADL-related indicators have almost no predictive ability for fall risk. Therefore, due to the higher risk of falls, individuals using psychotropic medications deserve attention from healthcare professionals, as well as from themselves and their families, and more detailed preventive measures should be implemented to reduce the risk of falls in this population.

Several recent studies have emphasized that fall prevention in older adults receiving psychotropic medications should be understood within the broader framework of individualized, risk-based prescribing and care^29,30^. Pastorino et al.^31^ highlighted that a sustainable and effective preventive strategy relies on precise identification of high-risk individuals and tailored interventions, rather than uniform approaches. Applied to our context, this supports not only examining psychotropic exposure itself (type, dose, and duration) but also integrating age, functional status, comorbidities, and hospitalization characteristics when designing fall-prevention strategies for psychotropic users. Austin et al.^32^ further argued that precision medicine in older adults must account for frailty, multimorbidity, polypharmacy, and trajectories of functional and cognitive decline, underscoring the need for nuanced deprescribing and monitoring decisions. In the setting of psychotropic-associated fall risk, this perspective reinforces the importance of individualized medication review, close monitoring of vulnerable subgroups, and dynamic adjustment of therapy. Taken together, these concepts align with our findings and suggest that psychotropic-related fall prevention should move toward a personalized, multidimensional approach rather than a one-size-fits-all strategy.

Although our study had a particular predictive effect on the risk of falls among the population using psychotropic medications, there are some limitations. Firstly, more detailed information regarding the types and duration of psychotropic medication use is needed. More cross-regional or interethnic collaborations are required, including more extensive prospective studies on the risk of falls among hospitalized patients. Furthermore, our study only recorded the occurrence of falls in the population without distinguishing the number of falls during the study period, which is also a limitation of the research. Additionally, the continual evolution of psychotropic medications may introduce heterogeneity between medication use and adverse-effect profiles at the time of the study and those at present. Meanwhile, in this dataset, fall events were identified using admission clinical records and a structured face-to-face questionnaire administered by nurses and physicians, which may miss unwitnessed or unreported events and could lead to outcome misclassification. Taken together, these limitations may bias our estimates, restrict generalizability, and thus warrant cautious interpretation of the findings.

## Conclusion

This study suggests that, compared with other inpatients in the cohort, patients receiving psychotropic medications have a higher risk of in-hospital falls. In this psychotropic medication subgroup, a multivariable model incorporating age, planned surgery, ADL feeding, and admission days showed moderate discriminatory ability for predicting falls. These findings indicate that healthcare professionals, patients using psychotropic medications, and their families should pay close attention to these risk factors and consider targeted fall-prevention strategies. At the same time, further external validation is needed before applying this model as a routine clinical tool.

## Electronic Supplementary Material

Below is the link to the electronic supplementary material.

Supplementary Material 1

## Data Availability

The dataset supporting this study is publicly available in the DRYAD digital repository under the accession number analysisdata_140723_3.sav.

## Abbreviations

ADL: Activities of daily living
OR: Odds ratio
MMT: Manual muscle test
IQIP: International Quality Indicator Project
