# Supplementary material for: Psychotropic medication and in-hospital falls in older adults: a cohort-based secondary analysis with exploratory stratification among users
Source: Sci Rep. 2025 Dec 6;16:642. doi: 10.1038/s41598-025-31320-7 (PMC12775001; doi:10.1038/s41598-025-31320-7)

**Supplementary Table 1.** Comparison between psychotropic medication group and non-psychotropic medication in the original inclusion population.

| Indicates | Group | PM Group (n=606) | Non-PM Group  (n=8863) | Z/X^2^ | P |
| --- | --- | --- | --- | --- | --- |
| Age |  | 58.0 (37.0, 72.0) | 63.0 (49.0, 73.0) | 5.078 | **<0.001** |
| Gender | Male | 233 | 4514 | 35.349 | **<0.001** |
|  | Female | 373 | 4349 |  |  |
| Falls | Yes | 43 | 187 | 59.495 | **<0.001** |
|  | No | 563 | 8676 |  |  |
| Wheelchair | Yes | 153 | 1715 | 12.447 | **<0.001** |
|  | No | 453 | 7147 |  |  |
| Needs help to move | Yes | 123 | 1219 | 19.953 | **<0.001** |
|  | No | 483 | 7643 |  |  |
| Rehabilitation | Yes | 8 | 50 | 8.426 | **0.015** |
|  | No | 579 | 8636 |  |  |
|  | Play doing | 18 | 173 |  |  |
| Laxative medication | Yes | 236 | 1428 | 203.830 | **<0.001** |
|  | No | 370 | 7429 |  |  |
| Remote carring system | Yes | 0 | 2 | 6.677 | 0.084 |
|  | No | 597 | 8793 |  |  |
|  | Crip censor | 6 | 30 |  |  |
|  | Other crip censor | 3 | 34 |  |  |
| Cognitive dysfunction | Yes | 50 | 187 | 87.623 | **<0.001** |
|  | No | 556 | 8674 |  |  |
| Eyesight | normal | 328 | 4623 | 3.658 | 0.600 |
|  | use glasses | 236 | 3459 |  |  |
|  | need help 1 | 26 | 462 |  |  |
|  | need help 2 | 13 | 281 |  |  |
|  | trance | 2 | 23 |  |  |
|  | zero | 0 | 4 |  |  |
| Sedative medication | Yes | 25 | 175 | 12.692 | **<0.001** |
|  | No | 581 | 8688 |  |  |
| Hypnotic medication | Yes | 345 | 1122 | 849.129 | **<0.001** |
|  | No | 261 | 7741 |  |  |
| Censor of bed | Yes | 9 | 66 | 3.063 | 0.080 |
|  | No | 597 | 8793 |  |  |
| Rihabiritation reviesed valuable | Yes | 26 | 223 | 7.007 | **0.008** |
|  | No | 579 | 8636 |  |  |
| Inhibition | Yes | 37 | 166 | 48.439 | **<0.001** |
|  | No | 569 | 8697 |  |  |
| Planned surgery | Yes | 429 | 3732 | 45.433 | **<0.001** |
|  | No | 171 | 5045 |  |  |
| History of falls | Yes | 121 | 707 | 102.746 | **<0.001** |
|  | No | 483 | 8147 |  |  |
| ADL sitting | normal | 513 | 7847 | 28.624 | **<0.001** |
|  | need help 1 | 6 | 106 |  |  |
|  | need help 2 | 30 | 164 |  |  |
|  | need help 3 | 14 | 195 |  |  |
|  | need help 4 | 42 | 531 |  |  |
|  | trance or zero | 1 | 15 |  |  |
| ADL Standing | normal | 482 | 7488 | 20.891 | **0.001** |
|  | need help 1 | 16 | 248 |  |  |
|  | need help 2 | 32 | 225 |  |  |
|  | need help 3 | 18 | 192 |  |  |
|  | need help 4 | 57 | 692 |  |  |
|  | trance or zero | 1 | 15 |  |  |
| ADLclothes | normal | 453 | 7236 | 18.882 | **0.002** |
|  | need help 1 | 10 | 86 |  |  |
|  | need help 2 | 68 | 691 |  |  |
|  | need help 3 | 30 | 346 |  |  |
|  | need help 4 | 44 | 488 |  |  |
|  | trance or zero | 1 | 13 |  |  |
| ADL feeding | normal | 488 | 7592 | 37.615 | **<0.001** |
|  | need help 1 | 12 | 75 |  |  |
|  | need help 2 | 46 | 299 |  |  |
|  | need help 3 | 11 | 143 |  |  |
|  | need help 4 | 48 | 737 |  |  |
|  | trance or zero | 1 | 14 |  |  |
| ADL toileting | normal | 460 | 7200 | 21.854 | **0.001** |
|  | need help 1 | 25 | 381 |  |  |
|  | need help 2 | 41 | 311 |  |  |
|  | need help 3 | 28 | 385 |  |  |
|  | need help 4 | 51 | 567 |  |  |
|  | trance or zero | 1 | 13 |  |  |
| ADL evacuation | normal | 456 | 7255 | 25.198 | **<0.001** |
|  | need help 1 | 20 | 282 |  |  |
|  | need help 2 | 47 | 360 |  |  |
|  | need help 3 | 39 | 451 |  |  |
|  | need help 4 | 43 | 499 |  |  |
|  | trance or zero | 1 | 13 |  |  |
| ADL wash face | normal | 461 | 7351 | 28.245 | **<0.001** |
|  | need help 1 | 16 | 194 |  |  |
|  | need help 2 | 67 | 539 |  |  |
|  | need help 3 | 22 | 324 |  |  |
|  | need help 4 | 39 | 437 |  |  |
|  | trance or zero | 1 | 14 |  |  |
| MMT Right foot | zero | 9 | 74 | 5.613 | **<0.001** |
|  | trance | 5 | 60 |  |  |
|  | poor | 9 | 66 |  |  |
|  | fair | 8 | 93 |  |  |
|  | good | 56 | 397 |  |  |
|  | normal | 517 | 8120 |  |  |
| MMT left foot | zero | 9 | 73 | 4.463 | **<0.001** |
|  | trance | 7 | 67 |  |  |
|  | poor | 6 | 81 |  |  |
|  | fair | 7 | 116 |  |  |
|  | good | 52 | 379 |  |  |
|  | normal | 522 | 8102 |  |  |
| Admission days |  | 12.0 (5.0, 24.0) | 10.0 (4.0, 19.0) | 3.049 | **0.002** |

PM: Psychotropic medication; ADL: Activities of daily living; MMT: Manual muscle test; Z: Non-parametric test result values; X^2^: chi-square test result values.

**Supplementary Table 2. The results of collinearity diagnosis (including ADL evacuation).**

| Model | | Unstandardized coefficient | | Standardized coefficient | t | Sig. | Collinearity statistics | |
| --- | --- | --- | --- | --- | --- | --- | --- | --- |
|  |  | B | SE | β |  |  | Tolerance | VIF |
|  | Constant | -0.032 | 0.084 |  | -0.378 | 0.706 |  |  |
|  | sex | -0.017 | 0.021 | -0.033 | -0.803 | 0.422 | 0.939 | 1.065 |
|  | age | 0.002 | 0.001 | 0.148 | 3.234 | 0.001 | 0.745 | 1.342 |
|  | wheelchair | -0.005 | 0.033 | -0.008 | -0.148 | 0.882 | 0.483 | 2.069 |
|  | needs help to move | 0.051 | 0.038 | 0.081 | 1.339 | 0.181 | 0.428 | 2.336 |
|  | planned surgery | -0.049 | 0.024 | -0.086 | -2.034 | 0.042 | 0.861 | 1.161 |
|  | history of falls | 0.052 | 0.027 | 0.083 | 1.931 | 0.054 | 0.840 | 1.191 |
|  | ADAclothesGroup | 0.074 | 0.049 | 0.126 | 1.510 | 0.132 | 0.223 | 4.492 |
|  | ADL wash face | 0.023 | 0.026 | 0.111 | 0.890 | 0.374 | 0.100 | **10.010** |
|  | ADL evacuation | -0.022 | 0.025 | -0.111 | -0.879 | 0.380 | 0.097 | **10.352** |
|  | MMT Right foot | 0.007 | 0.019 | 0.025 | 0.389 | 0.698 | 0.379 | 2.639 |
|  | MMT Left foot | -0.010 | 0.019 | -0.034 | -0.526 | 0.599 | 0.373 | 2.680 |
|  | admission days | 0.002 | 0.001 | 0.175 | 4.286 | 0.000 | 0.936 | 1.068 |
|  | laxative medication | -0.003 | 0.022 | -0.006 | -0.153 | 0.879 | 0.857 | 1.167 |
|  | cognitive dysfunction | -0.001 | 0.040 | -0.002 | -0.036 | 0.971 | 0.825 | 1.211 |
|  | eyesight | -0.033 | 0.015 | -0.092 | -2.183 | 0.029 | 0.874 | 1.144 |
|  | sedative medication | 0.015 | 0.055 | 0.011 | 0.276 | 0.783 | 0.911 | 1.097 |
|  | hypnotic medication | 0.010 | 0.022 | 0.019 | 0.456 | 0.649 | 0.875 | 1.142 |
|  | ADL Feeding | -0.034 | 0.017 | -0.164 | -2.035 | 0.042 | 0.239 | 4.183 |

SE: Standard error; a. Dependent variable: fall event; VIF: Variance Inflation Factor.

**Supplementary Table 3. The results of collinearity diagnosis (excluding** **ADL evacuation).**

| Model | | Unstandardized coefficient | | Standardized coefficient | Unstandardized coefficient | Sig. | Collinearity statistics | |
| --- | --- | --- | --- | --- | --- | --- | --- | --- |
|  |  | B | SE | β |  |  | Tolerance | VIF |
|  | Constant | -0.047 | 0.082 |  | -0.573 | 0.567 |  |  |
|  | sex | -0.018 | 0.021 | -0.035 | -0.852 | 0.395 | 0.942 | 1.062 |
|  | age | 0.002 | 0.001 | 0.148 | 3.233 | 0.001 | 0.745 | 1.342 |
|  | wheelchair | -0.004 | 0.033 | -0.006 | -0.111 | 0.912 | 0.484 | 2.066 |
|  | needs help to move | 0.046 | 0.038 | 0.073 | 1.228 | 0.220 | 0.437 | 2.290 |
|  | planned surgery | -0.049 | 0.024 | -0.087 | -2.042 | 0.042 | 0.861 | 1.161 |
|  | history of falls | 0.051 | 0.027 | 0.080 | 1.867 | 0.062 | 0.845 | 1.183 |
|  | ADLclothes Group | 0.065 | 0.048 | 0.110 | 1.354 | 0.176 | 0.233 | 4.285 |
|  | ADL wash face | 0.009 | 0.020 | 0.041 | 0.427 | 0.670 | 0.169 | 5.903 |
|  | MMT Right foot | 0.009 | 0.019 | 0.029 | 0.450 | 0.653 | 0.381 | 2.627 |
|  | MMT Left foot | -0.008 | 0.019 | -0.026 | -0.401 | 0.689 | 0.381 | 2.621 |
|  | admission days | 0.002 | 0.001 | 0.173 | 4.260 | 0.000 | 0.938 | 1.067 |
|  | laxative medication | -0.004 | 0.022 | -0.008 | -0.182 | 0.856 | 0.858 | 1.165 |
|  | cognitive dysfunction | -0.003 | 0.040 | -0.003 | -0.076 | 0.939 | 0.827 | 1.209 |
|  | eyesight | -0.033 | 0.015 | -0.092 | -2.191 | 0.029 | 0.874 | 1.144 |
|  | sedative medication | 0.013 | 0.055 | 0.010 | 0.240 | 0.811 | 0.913 | 1.096 |
|  | hypnotic medication | 0.010 | 0.022 | 0.019 | 0.447 | 0.655 | 0.875 | 1.142 |
|  | ADL Feeding | -0.037 | 0.016 | -0.179 | -2.264 | 0.024 | 0.250 | 4.007 |

**Supplementary Figure 1. LASSO Logistic Regression: CV Curve.**


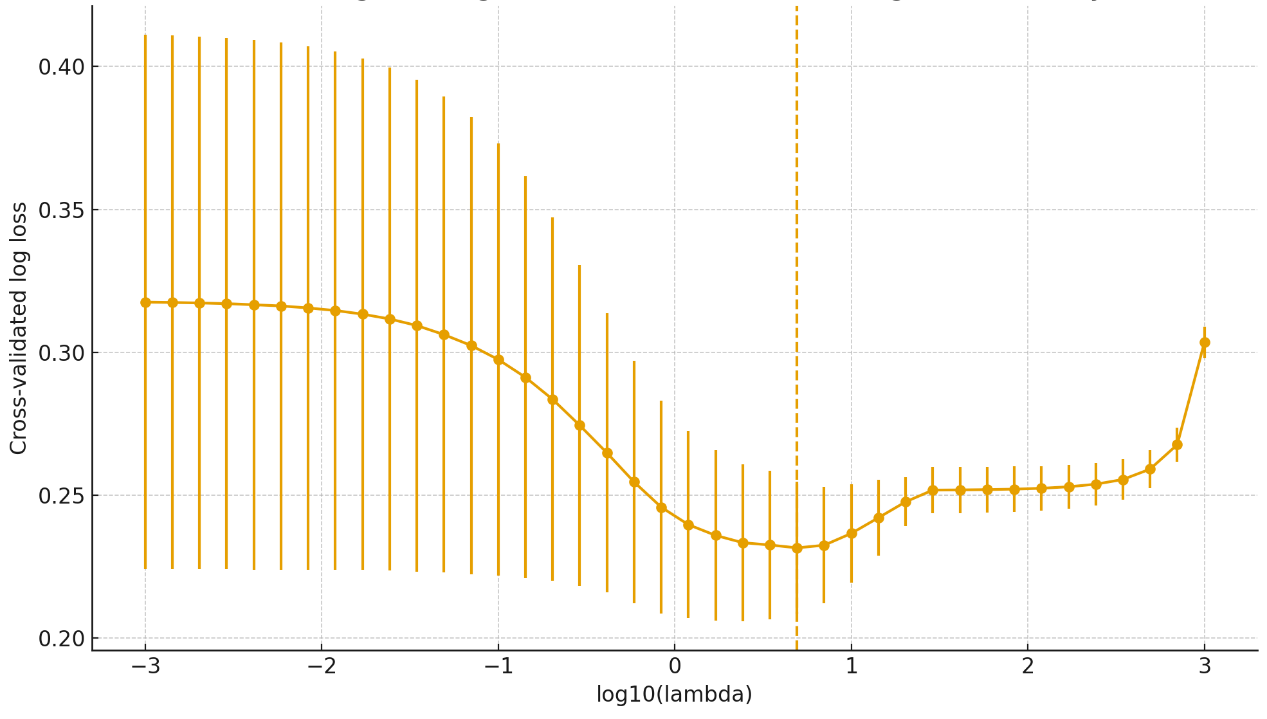

Supplement: Supplementary file 1 — Supplementary Material 1 [file 41598_2025_31320_MOESM1_ESM.docx]
